# Environmentally Friendly Valorization of *Solieria filiformis* (Gigartinales, Rhodophyta) from IMTA Using a Biorefinery Concept

**DOI:** 10.3390/md16120487

**Published:** 2018-12-06

**Authors:** Ana Peñuela, Daniel Robledo, Nathalie Bourgougnon, Gilles Bedoux, Emanuel Hernández-Núñez, Yolanda Freile-Pelegrín

**Affiliations:** 1 Marine Resources Department, CINVESTAV-Mérida, A.P. 73, Cordemex, Mérida, Yucatán 97310, Mexico; ana.penuela@cinvestav.mx (A.P.); daniel.robledo@cinvestav.mx (D.R.); emanuel.hernandez@cinvestav.mx (E.H.-N.); 2 Laboratoire de Biotechnologie et Chimie Marines, Université de Bretagne Sud, EA3884, UBS, IUEM, F-56000 Vannes, France; nathalie.bourgougnon@univ-ubs.fr (N.B.); gilles.bedoux@univ-ubs.fr (G.B.)

**Keywords:** *Solieria filiformis*, biorefinery, carrageenan, antiviral activity, IMTA, MAE, EAE

## Abstract

Marine macroalgae (seaweed) are an excellent source of novel bioactive metabolites. The biorefinery concept applied to seaweed facilitates the extraction of many chemical constituents from the same biomass ensuring that the resource is used fully, generating few residues through a succession of extraction steps. In the present study, the biomass of the carragenophyte *Solieria filiformis* (Rhodophyta, Gigartinales) cultured in an integrated multi-trophic aquaculture (IMTA) system was evaluated to obtain valuable products by a biorefinery approach. Enzymatic-assisted extraction (EAE) and microwave-assisted extraction (MAE) were the eco-friendly technologies used to ensure an environmentally friendly valorization of the biomass. Three valuable products were successfully recovered: a water-soluble extract rich in proteins and sulfated polysaccharides suitable as a food supplement; a lipid fraction rich in polyunsaturated fatty acids (PUFAs) with potential to be used in the nutraceutical industry; and a pure ι-carrageenan with a powerful antiviral activity against *Herpes simplex* virus (EC_50_ = 6.3 µg mL^−1^) comparable to the commercial antiviral acyclovir (EC_50_ = 3.2–5.4 µg mL^−1^).

## 1. Introduction

Seaweeds are mainly used as raw material for human nutrition and for the phycocolloid industry. Because they live in complex habitats, they are excellent sources of new bioactive metabolites that cannot be found in other organisms. According to their pigment composition seaweeds are classified into Rhodophyta, Phaeophyceae, and Chlorophyta (red, brown and green seaweeds respectively). Several species of Rhodophyta are economically important resources due to their production by cultivation [1,2], which represents 61.3% (≈18 million tonnes) of the total global seaweed production worldwide [3]. Red seaweeds are mainly used to extract agar and carrageenan, two phycocolloids used as thickeners and gelling agents in food and cosmetic industries. Rhodophyta have also been gaining considerable importance as a source of other valuable compounds of nutritional and nutraceutical importance such proteins and polyunsaturated fatty acids (PUFAs) [4]. Furthermore, carrageenans have also shown different biological activities such as antitumoral, antioxidant, and antiviral against a broad spectrum of viruses including *Herpes simplex* virus, which affects around 67% of the world population [5].

Carrageenans are a cell wall sulfated galactans family that comprised of alternating 3-linked β-d-galactose (G units) and 4-linked α-d-galactose (D units) or 4- linked 3,6-anhydro-d-galactose (A units). Depending of the number and position of sulfate groups, carrageenans are divided into different types: kappa (κ-; G4S-DA), iota (ι-; G4S-DA2S), and lambda (λ-; G2S-D2S,6S) [6]. Minor types are mu (μ-; G4S-D6S) and nu (υ-; G4S-D2S6S), which are κ- and ι-precursors, respectively. The complexity of cell wall in Rhodophyta is a major problem when extracting metabolites. The presence of large quantities of interconnected polysaccharides (i.e., carrageenans), as well as proteins, reduces the efficiency of the standard extraction methods used to date [7]. Furthermore, most of these methods generate chemical pollution through the use of large amounts of solvents in processing, which is also time consuming [8]. Recently, other alternatives have been proposed to reduce time and/or waste through ecofriendly extraction technologies [9,10]. Enzyme-assisted extraction (EAE) and microwave-assisted extraction (MAE) are some of the environmentally friendly techniques that are being used to improve biological activities of certain metabolites [8,11]. MAE is one of the most successful extraction processes for the isolation of bioactive compounds from marine macroalgae, and to obtain extracts rich in sulfated polysaccharides that exhibit different biological activities [12].

Additionally, the production cost of novel valuable compounds from seaweeds is still high. Extracting many compounds either at the same time or successively in a biorefinery concept could compensate for this fact. An integrated sustainable biorefinery approach facilitates the extraction of many chemical products from the same biomass, maximizing its value and generating fewer residues through a succession of extraction steps [9,13]. Under the biorefinery concept, various models have been proposed in seaweeds to deliver a diverse range of products, which generally end up with phycocolloids, once all other valuable components have been removed. Thus, a biorefinery approach in carragenophytes should be based on the production of carrageenan as the main extraction compound since it is already an industrialized and mature technology [10,14]. Until now there have been no studies about the biorefinery process being applied to carragenophytes as the feedstock.

Another important aspect to take into account is the way the seaweed biomass is produced. The use of aquaculture wastewater as a nutrient feed for seaweeds is recognized as the most promising proposal for reducing the negative impacts of aquaculture in the surrounding environment, as well as a way to obtain algae biomass. Integrated multi-trophic aquaculture (IMTA) systems are based on the use of organisms from different trophic levels where the excretion of upper-level organisms becomes a resource utilized by the lower levels. In an IMTA system, the mitigation of the potential impacts of these excretions is performed mainly by lower trophic levels, primarily macroalgae, due to their biofiltration capacity that improves water quality by absorbing dissolved nutrients [1,15]. This reduces the cost of water treatment and creates a new crop with great potential economic value [16,17,18]. Thereby, the concept of ecological aquaculture or bioextraction to obtain algal biomass, coupled with the use of environmentally friendly techniques and the biorefinery concept, could be a research strategy focus into the ‘Blue Economy’ that highlights the sustainable use of oceanic resources by providing economic and ecological benefits.

Among the current commercially exploited carragenophytes, the most abundant genus belongs to the Solieriaceae family (Gigartinales). *Solieria filiformis* (Kützing) Gabrielson has been reported as a source of relatively pure ι-type carrageenan, although a high number of different structural elements, including both ι- and κ-carrageenans, can be found depending on the biomass. Bioprospecting activities and previous biochemical studies revealed that this species also contains several valuable bioactive compounds [19,20,21,22]. In the light of the abovementioned results and in the need of environmentally friendly valorization of algae biomass, the aim of this study was to assess the potential of *Solieria filiformis* produced in an IMTA system to obtain three valuable products (a water- soluble extract, fatty acids and carrageenan) through a biorefinery approach using EAE and MAE as eco-friendly extraction technologies.

## 2. Results and Discussion

### 2.1. Solieria filiformis Biomass Production under an Integrated Multi-Trophic Aquaculture (IMTA) System

*Solieria filiformis* has been grown successfully under an IMTA system. The growth performance of *S. filiformis* cultured in the IMTA system and in the control unit (cultured only with seawater) are presented in Table 1. *S. filiformis* growth rates and productivity under IMTA conditions were higher, twice and 2.6 fold, respectively, when compared to the control unit. Moreover, these values were comparable, or even higher, to those reported for other Rhodophyta growing in IMTA systems [17,23,24,25,26].

No significant differences were observed in C:N ratios in algal tissue from *S. filiformis* cultured in IMTA and in the control unit. This suggests that *S. filiformis* possess a high nitrogen storage capacity under different nutrient conditions that allows it to grow and use it for its metabolic maintenance. This can also be corroborated by the observed ammonium removal efficiency (ARE), which was similar (~40%) for algae grown at both systems, although ammonium input was higher in the IMTA system due to the nutrients released by fish and sea cucumbers (31.8 ± 16.0 in the IMTA system versus 25.8 ± 14.9 μM NH_4_^+^ in the control unit). The sources of nitrogen input in an aquaculture system are metabolic excretions and non-consumed feed. Ammonium is considered the main product of nitrogen metabolism, and consequently is the cheaper form of nitrogen excretion [27]. In fish and other organisms, these metabolites must not remain in the cells and tissues due to their high toxicity, and therefore it is advisable to remove them from the culture system. In the fish and sea cucumber culture tanks, ammonium production was continuous and the macroalgae was able to assimilate it. In the present study, the ARE of *S. filiformis* was similar to that reported for Gracilaria vermiculophylla [28], but lower than those reported for *Hydropuntia cornea* (≥60%) [17]. In order to optimize the nutrient removal efficiency and to maximize the biofiltration capacity of *S. filiformis* several factors must be carefully regulated (i.e., nutrient supply, flow rates), therefore other studies should be performed on this subject.

It is also interesting to note that epiphyte content (filamentous algae and invertebrates) was significantly lower in *S. filiformis* grown in the IMTA system when compared to that cultivated only with seawater. Moreover, algae from IMTA system showed a deeper red color and a healthier appearance than those in the control unit. The increase in epiphytes in seaweeds grown only with seawater may indicate lower physiological conditions in *S. filiformis*, allowing the overgrowth of opportunistic algae on the thalli. This difference could be related to the fact that a significant fraction of the nitrogenous waste being was converted relatively faster into biomass under IMTA conditions, limiting epiphytic growth [29].

From the above results, it can be concluded that *S. filiformis* cultured in an IMTA system exhibits a better growth performance to obtain clean and healthy biomass for useful marketable algae biomass. In addition, this species has the potential to mitigate the environmental impacts of aquaculture by improving water quality. In the light of the above promising results, *S. filiformis* biomass obtained from IMTA culture was used in this study for its valorization using a sequential extraction process with a biorefinery approach.

### 2.2. Initial Proximate Composition of Solieria filiformis

Results on the proximate composition of *S. filiformis* obtained from the IMTA system and from the control unit provide basic information to determine the potential utilization of this species (Table 2).

In general, dry weight and carbohydrate content were similar to those reported for other *Solieria* species [7,21]. *S. filiformis* from IMTA showed higher dry weight values than biomass from the control, corroborating the higher growth rate found in the IMTA system. Total protein content was lower than previously reported for this species (15–20%) [21,30,31], while the lipid content was higher to other reports [31], and slightly higher from the IMTA biomass when compared to the control unit. In relation to the sulfate content, Morán-Santibañez et al. [20] found significant lower sulfate content (7.5 ± 0.5%) than those reported in the present study. From our results, the higher content of carbohydrates and sulfates found in the algae from the IMTA system could be related to an overall increase in the sulfated polysaccharides. In many Rhodophyta, these polysaccharides have been associated as inducers of a defense response against pathogens [32]. *S. filiformis* in the IMTA system was fed with discharges water from fish and sea cucumbers, which may contain a high load of microorganisms, and thereby the increase in sulfate content could be related to a defense mechanism in accordance with the aforementioned.

### 2.3. Products Obtained from the Sequential Extraction Process and Their Quantitative and Qualitative Analysis

#### 2.3.1. Recovery of Water-Soluble Extract (WSE) by Biorefinery Process ant Its Potential Application (**Product 1**)

In general, yields obtained using the enzyme (63.5 and 72.4%, conditions a and b respectively) increased in comparison to the blanks (without enzyme) which were 58.6% and 47.6%, showing a 24% gain for **Product 1*a*** and 33% for **Product 1*b***. The yield and biochemical composition of water-soluble extract WSE obtained by EAE (**Product 1*a*** and **Product 1*b***) and their corresponding residues (Residue 1***a*** and Residue 1***b***) are presented in Table 3. For the denatured enzyme extraction (***a***), the yield was higher than that under non-denatured enzyme extraction (***b***), and it was also higher than that reported for *S. chordalis* (55.8%) using the same EAE conditions [7]. A similar pattern was observed for carbohydrate and sulfate contents, with higher values obtained in **Product 1*a*** when compared to **Product 1*b*** (31.9 and 26.1% higher, carbohydrates and sulfates respectively). No significant differences in protein and lipid content were observed between the two conditions tested. The increase in temperature to 85 °C for 15 min to denature the enzyme allowed a higher yield of the polysaccharide. In relation to this, higher extraction efficiencies of sulfated polysaccharides, such as carrageenan, has been observed and widely reported at high temperatures [11,33,34,35]. The residues from the EAE extraction step generated under different conditions, residues 1***a*** and 1***b***, rich in carbohydrates (~32%) and sulfate (~23%), were used to obtain **Product 2**.

The cytotoxicity and in vitro antiviral activity of **Products 1*a***–***b*** are presented in Table 4. No cytotoxic effect on the Vero cells was observed in the range of the concentrations assayed. After 3 days of treatment, no microscopically visible alteration of normal cell morphology was observed and a viability assay showed no destruction of cell layer. Only **Product 1*a*** exhibited antiviral activity against *Herpes simplex* virus. In *S. chordalis* a similar EC_50_ value (86.0 μg/mL) was reported using the same EAE conditions [7]. The higher carbohydrate and sulfate contents exhibited by **Product 1*a*** in comparison with **Product 1*b*** (Table 3), its probably related with a higher carrageenan proportion that could be responsible for this activity. It has been previously reported that sulfated polysaccharides, including carrageenans, have antiviral activities against enveloped viruses such as dengue virus, papillomavirus and *H. simplex* [20,36], where the degree of sulfation is the main factor related to the antiviral activity [37]. Many biological effects of sulfated polysaccharides are related to their ability to change the surface properties of the cell, which interferes with the early events that impede the entry of the virus since these compete with similar molecular species for initial binding to the target cell [38].

The selectivity index (SI) was used to compare the antiviral potency of the extracts. Despite the high antiviral activity shown by **Product 1*a***, weak antiviral potency (<10) was obtained (Table 4). However, it should be noted that this is a crude extract rich in sulfated polysaccharides, and this activity could be improved by adding a purification step to obtain a pure carrageenan.

#### 2.3.2. Recovery of Fatty Acids (FAs) by Biorefinery Process and its Potential Application (**Product 2**)

From the EAE Residues 1***a*** (19.9%) and Residue 1***b*** (22.5%) the lipids obtained (~0.71%) were used as raw material to obtain FAs (**Product 2**). The fatty acids (FAs) profile and nutritional indices (ω6/ω3 and saturated/unsaturated fatty acids (SFAs/UFAs)) of **Products 2*a***–***b*** are shown in Table 5. The FAs profile obtained from the initial *S. filiformis* raw biomass by direct extraction is also included for comparison.

The FAs profile obtained for *S. filiformis* in this study was similar to those reported for other *Solieria* species in previous studies [39]. A high content of SFAs (69.2–73.6%) were obtained for both **Product 2*a*** and **Product 2*b***, as well as for FAs obtained directly from raw material, without significant differences among them. Palmitic acid C16:0 was the most abundant SFA (43.9–55.8%) in all conditions. Although from a nutritional point of view dietary SFAs are usually associated with negative consequences for human health, the role of individual SFAs in metabolic disease is still incompletely understood. Individual SFAs such as myristic (C14:0) and palmitic acids, both present in *S. filiformis*, are involved in protein fatty acid acetylation, which may have various regulation functions and, as has been recently reviewed, in the future dietary recommendations will probably evolve to differentiate them [40,41].

Concerning unsaturated FAs (UFAs), MUFAs content in **Products 2*a***–***b*** ranged from 12.47 to 16.57% and no significant differences were found between condition ***a*** and ***b***. However, there was a significant reduction of MUFAs when compared with those obtained directly from raw material. Palmitoleic acid (C16:1n-7) and oleic acid (C18:1n-9c) were present in the MUFAs fraction. Healthy diets are abundant in MUFAs that are often associated with low prevalence of chronic disease. These include oleic acid that has been reported to reduce cardiovascular risk by reducing blood pressure [42,43], and also palmitoleic acid that has been recently reported to have anti-inflammatory and antidiabetic activities [44].

PUFAs corresponded to ~14% in **Products 2*a***–***b*** and, contrary to MUFAs, they almost doubled their content when compared with those obtained directly from raw material. Eicosapentaenoic acid (EPA, C20:5 ω3) and arachidonic acid (AA, C20:4 ω6) were detected in **Product 2*b*** as well as in FAs obtained directly from *S. filiformis*, whereas the presence of γ-Linolenic acid and Linolenic acid (C18:3 ω6 and C18:2 ω6, respectively) were only observed in FAs obtained directly from *S. filiformis*. PUFAs have a great importance for human metabolism. They are the major components of cell membrane phospholipids, and may also be present in cellular storage oils [45,46]. Both AA and EPA are two crucial fatty acids of marine origin. AA is the principal ω6 fatty acid in the brain development of infants, and EPA has a beneficial effect on the cardiovascular system. They are also precursors of prostaglandins, thromboxane and other eicosanoids involved in immune reactions. Because their beneficial properties such as antibacterial, anti-inflammatory, antioxidant, antitumoral properties and the prevention of cardiac diseases, they are important nutraceutical and pharmaceutical targets [47]. The absence of AA and EPA in **Product 2*a*** could be related to the increase of temperature (85 °C for 15 min) to denature the enzyme in that condition. Although the applications of enzymes in bioprocessing are especially advantageous because they act under mild reaction conditions, it has been reported that PUFAs are highly labile to pH or elevated temperatures that can produce an oxidation or double bond migration [48].

As for the ω6/ω3 ratio, based on the FA results it was calculated at 0.91 for **Product 2*b*** and 5.01 for the control. The World Health Organization (WHO) currently recommends that the ω6/ω3 ratio should not exceed 10 to have protective effects against atherosclerotic heart disease, and in the prevention of chronic diseases [49].

In the same regard, the SFAs/UFAs ratio is also an indicator used to evaluate the quality of lipids in a nutritional sense, since UFAs are essential for the proper functioning of the human body. The balance of SFAs with UFAs, especially PUFAs, has been associated with a lower risk of heart diseases. Although **Products 2*a***–***b*** displayed high amounts of SFA, the high content of MUFAs and PUFAs indicates a potential nutritional value. Moreover, the increase in the relative abundance of PUFAs observed after the enzymatic extraction raises the potential value of this product once it enters into the biorefinery process.

#### 2.3.3. Recovery of Carrageenan by Biorefinery Process and Its Potential Application (**Product 3**)

In the third stage of the sequential extraction process, residual biomass (Residues 2***a***–***b***) resulted from lipid extraction (~96%) was further processed for the recovery of *S. filiformis* carrageenan by MAE. Yield and chemical characterization of carrageenan (**Products 3*a***–***b***) are shown in Table 6.

Carrageenan yields obtained at the end of the biorefinery process of *S. filiformis* ranged between 17.1 to 29.7%, whereas that obtained by direct extraction was 25.5%, all of them were in accordance with yields reported for *Solieria chordalis* by a direct extraction using MAE [8]. Our results were also comparable, or even higher, to those reported previously by Fernandes de Araujo et al. (19.1%) [19], and Caamal-Fuentes et al. (17.8%) [22] for *S. filiformis* using direct carrageenan extraction by conventional methods, where higher carrageenan yields should be expected because no losses related to a sequential extraction process. Moreover, in our case MAE favored the extraction of carrageenan due to the high temperature reached using closed vessels, increasing the mass transfer of carrageenan from the sample matrix. The efficiency of this method over conventional extraction methods has already been previously reported [8,11].

**Figure 1:**
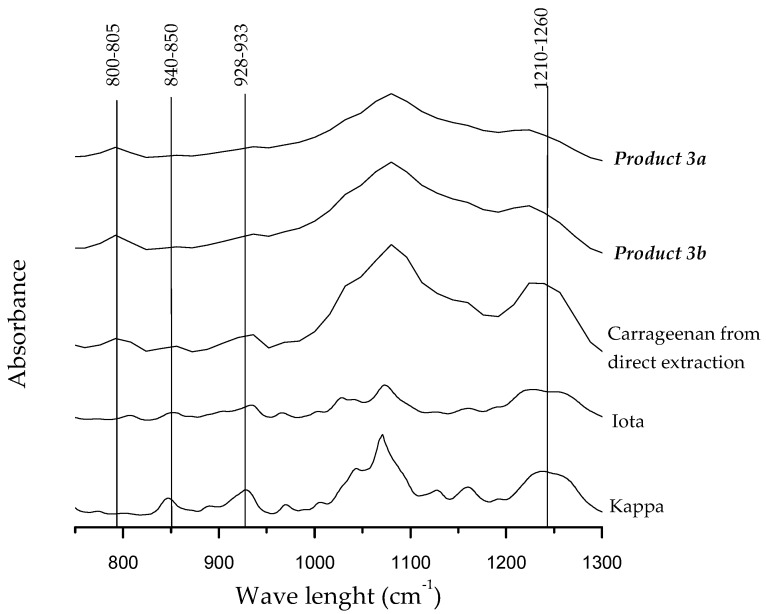
Fourier transform–infrared (FT–IR) spectra of carrageenans from *Solieria filiformis* extracted by MAE. ***a*** and ***b*** correspond to conditions denatured and non-denatured enzyme, respectively.

It is noteworthy that carrageenan yields and their chemical properties were significantly influenced by the extraction conditions of the first step during the sequential extraction process of *S. filiformis* in relation with the denatured and non-denatured enzyme. The lowest carrageenan yield, as well as lowest sulfate content, was obtained in **Product 3*a*** (Table 6). During EAE extraction conditions ***a*** and ***b*** (corresponding to denatured and non-denaturated enzyme respectively), the high temperatures applied to denature the enzyme together with elevated temperatures reached by microwave heating could explain these differences. Clearly, cell wall maceration during enzymatic hydrolysis treatments could potentially increase the effect of MAE in the subsequent extraction processes. However, in the present study, the use of this pre-treatment before carrageenan extraction did not significantly improve the yield. This behavior can be partly explained due to a degradation of polysaccharides by an excessive thermal treatment and the release of oligosaccharides resulting in lower carrageenan yield in **Product 3*a***. Additionally, variability using different enzymes in *Solieria chordalis* extraction treatments has been previously reported [7,50]. This effect, due not only to the thermal treatment itself but also to the specific aggressiveness of the enzyme, could also be related to the lower yields obtained in the present study under extraction condition ***a***. In this way, to better understand the effect of the different parameters involved in a sequential carrageenan extraction process, further studies on the efficacy of other enzymes to optimize the extraction capacity should be performed. In this study, sulfate and 3,6 anhydro-galactose content were in the range to those previously reported in *S. filiformis* [20,22]. The Fourier transform–infrared (FT–IR) spectra are shown in Figure 1.

The presence of ι-carrageenan in **Products 3*a***–***b*** and in carrageenan obtained by direct extraction was detected with absorption bands observed at 800–805 and 840–850 cm^−1^ characteristic for DA2S and G4S respectively (Figure 1). Typical absorption bands at 1210–1260 cm^−1^ common to all types of compounds containing sulfate, and one band at 930 cm^−1^ specific to 3,6-anhydro-d-galactose were also present [51]. Bands at 825 and 867 cm^−1^ specific of the precursor μ- and ν-linked to C6 (D6S) were not observed.

**Figure 2:**
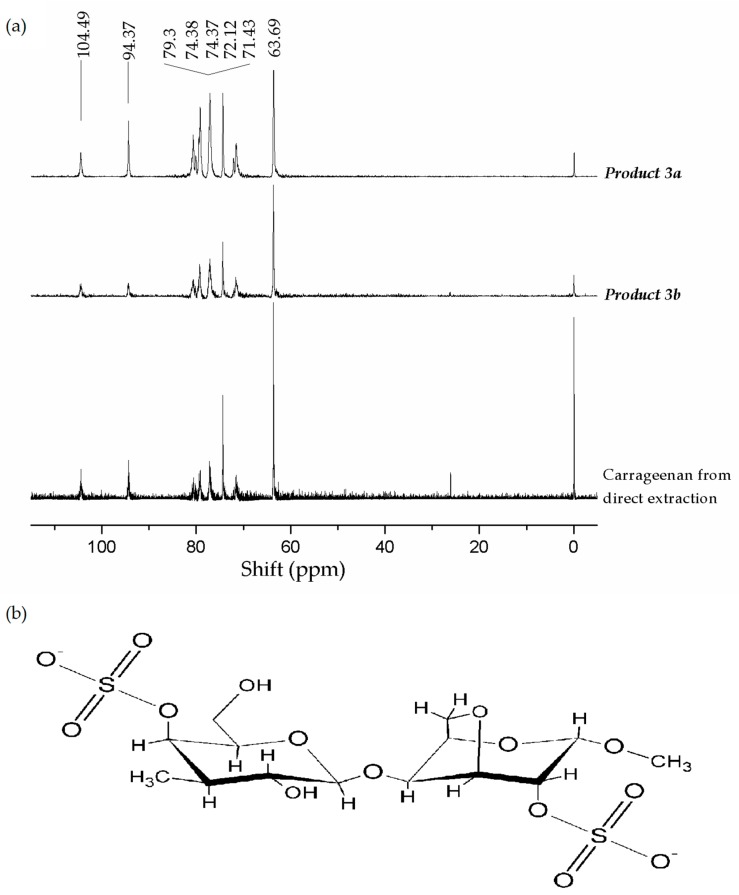
(**a**) ^13^C nuclear magnetic resonance (NMR) of carrageenans from *Solieria filiformis* operating at frequencies of 150.81 MHz for ^13^C nucleus. Iota carrageenan characteristic signal of anomeric carbon (C1) of G4S correspond at δ 104.49 ppm and anomeric carbon (C1) DA2S correspond at 94.37 ppm. (**b**) Chemical structure of ι-carrageenan.

The ι-carrageenan structure in **Products 3*a***–***b*** and in carrageenan obtained by *S. filiformis* direct extraction was confirmed by ^13^C nuclear magnetic resonance (NMR) spectra (Figure 2). Carbohydrates signals were noted at 63.69 and 104.49 ppm. Typical signals of ι-carrageenan for anomeric carbon (C1) of G4S at 104.49 ppm and anomeric carbon (C1) of DA2S at 94.37 ppm [52] were observed. Other typical signals of G4S at 63.69, 71.43 and 74.38 ppm attributed to carbon C6, C2 and C4 were observed. Peaks at 72.12, 74.37 and 79.3 ppm of DA2S attributed to C2, C4 and C5 were also detected [8,21,22,52]. Characteristic signals of anomeric carbons for other polysaccharides or for the μ- and ν-precursors were not detected, indicating the presence of a fairly pure ι-carrageenan.

The cytotoxicity and in vitro antiviral activity of **Products 3*a***–***b*** and those obtained from carrageenan by direct extraction are presented in Table 7. Similar to the results obtained from **Products 1*a***–***b*** (water-soluble extracts), no cytotoxic effect on the Vero cells was observed in the range of the concentrations assayed. After 3 days of treatment, no microscopically visible alteration of normal cell morphology was observed and viability assay showed no destruction of the cell layer. All carrageenans (**Products 3*a***–***b*** and that obtained from direct extraction) exhibited a powerful antiviral activity against *Herpes simplex* virus, higher to that showed by **Product 1*a***, and very close to that showed by acyclovir.

It is noteworthy that the strongest antiviral activity was obtained by **Product 3*b***, which showed the highest sulfate content and lowest 3,6-anhydro-galactose (34.9% and 7.43% for sulfate and 3,6-anhydro-galactose, respectively). As mentioned above, previous studies have shown that degree of sulfation has a major impact on the antiviral activity of sulfated polysaccharides because of its ability to interfere with the initial attachment of the virus to the target cell [53,54]. Recently a synergistic antiviral effect of sulfated polysaccharides extracted from the brown seaweed Eisenia arborea and *S. filiformis* on the enveloped measles virus has been reported [20]. Authors reported that the most efficient inhibition was obtained in the early phases of infection (0 and 15 min after infection). Furthermore, it has been suggested that the conformational flexibility of the binding region plays an important role in the antiviral activity of sulfated polysaccharides, and the chain flexibility of random coil carrageenans increases with the percentage of 3,6-anhydro-galactose [55,56]. According to our results, the lower 3,6-anhydro-galactose content found in **Product 3*b*** would indicate that a decrease in chain flexibility could be related to an increase in the antiviral activity. However, besides the degree of sulfation other factors seems to be involved in the formation of the polysaccharide-virus complex such as the molecular weight and the distribution of sulfate groups [54]. In this way, more chemical and structural analyzes of this promising carrageenan should be carried out, as well as studies on its mechanism of action as an antiviral agent.

### 2.4. Integrated Process Performance

*Solieria filiformis* was successfully integrated into a sustainable aquaculture system (IMTA) obtaining high productivity and growth rates that allowed us to obtain a clean and healthy biomass. In addition, this species showed a potential capacity to mitigate the environmental impacts of aquaculture by improving water quality due to its biofilter capacity. The biomass produced was used as a feedstock to develop a valorization strategy of the species to obtain valuable products using a cascading biorefinery approach by the assistance of green technologies using EAE and MAE. These eco-friendly techniques allowed us to obtain high extraction yields that represent an advantage for sustainable development. However, a technological target to take into account is to optimize conditions during the EAE (i.e., testing different enzymes) to extract higher quantity and quality of valuable products.

Although the focus was to obtain carrageenan as the main product from *S. filiformis*, our results contribute towards an integrated biorefinery approach for maximizing the whole biomass value. The global carrageenan industries process 202,500 dry tons of carrageenophytes annually to produce 65,000 tons of carrageenan [57], and the remainder is lost as waste. However, processing of that biomass using a biorefinery model can lead to the recovery of a number of products along with tons of carrageenan. In the present study, the process developed successfully recovered 3 products of commercial value: a WSE rich in nutrients suitable as a food supplement and with potential antiviral activity against *Herpes simplex* virus type 1; a lipid fraction rich in PUFAs with potential use in the nutraceutical industry; and a pure ι-carrageenan with potential applications as antiviral against *H. simplex*.

Table 8 summarizes yields and quality parameters of *S. filiformis* products obtained by direct extraction and by integrated biorefinery process.

From Table 8 it can be inferred that from 1 ton of dry biomass of *S. filiformis*, 700 kg of antiviral compound, 600–700 g of PUFAs, and 170–300 kg of ι-carrageenan with a powerful antiviral activity can be recovered by the biorefinery approach used. This process generates 6.3–10.4% of residues from the initial biomass, while the residues obtained from a direct extraction are 15 times higher. Despite these promising results and as a next step, the feasibility of the proposed biorefinery based on *S. filiformis* obtained from an IMTA system should also be evaluated through an analysis of the economic and environmental system.

## 3. Materials and Methods

### 3.1. Solieria filiformis Biomass Production under an IMTA System

*Solieria filiformis* is currently cultivated by our group in a land-based outdoor culture system located at the Cinvestav Coastal Marine Station at Telchac, Yucatán, México (21°20′28′ N, 89°18′25′ W). The ‘seeding’ for the production of the *S. filiformis* biomass used in the experimental IMTA system was supplied from this culture.

#### 3.1.1. Experimental IMTA System

**Figure 3:**
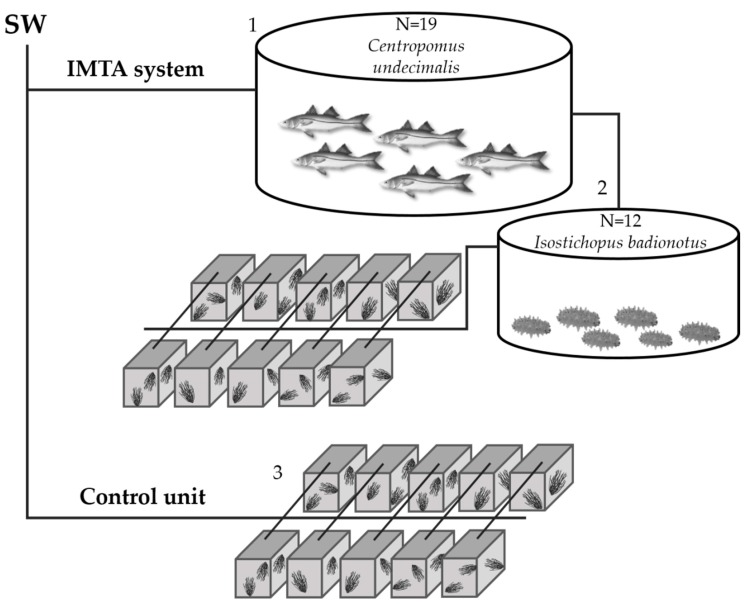
IMTA system design: Fish tank (1) connected to sedimentation tank with sea cucumber (2). Wastewater effluents from sedimentation tank were connected to *S. filiformis* tanks (3). Control unit only supplied with clean seawater (SW).

The IMTA system designed for the present study is described as follows: a circular 2000 L tank was stocked with 19 adult snooks (*Centropomus undecimalis*). The effluent from this tank was connected to a circular 700 L tank used as sedimentation tank to trap particulate organic matter. In the sedimentation tank, 12 chocolate chip sea cucumbers (*Isostichopus badionotus*) were placed to feed with the excess organic matter generated. Finally, the effluent from these tank were used to cultivate *S. filiformis* at a density of 3 g fresh weight L^−1^. *Solieria filiformis* vegetative fragments were placed into 10 replicate 50 L containers supplied with a continuous flow of wastewater from the sedimentation tank (Figure 3). All tanks and containers were continuously aerated. An experimental unit receiving only a continuous flow of clean seawater (SW) was used as control. The experimental period consisted of 5 weeks (two for acclimation and three for collecting the data). The total ammonium mean concentration (μM) at the inflow and outflow of the *S. filiformis* cultivation system was measured during all the experiment period. ARE was calculated as a percentage of the inputs calculated following the equation:

ARE (%) = 100 − (100 × At/A_0_) (1)

where A_0_ and At are ammonium input and output, respectively.

*Solieria filiformis* biomass was harvested weekly to maintain the initial stocking density. Biomass collected was washed with freshwater to remove mud and salts, freeze-dried and stored until further analysis. Before restocking the tanks, sub-samples of *S. filiformis* were thoroughly cleaned to assess the epiphyte content. Between each harvest period, the tanks were emptied and cleaned.

#### 3.1.2. Calculations of Algal Growth Parameters

Seaweed specific growth rate was determined weekly using the formula:

% day^−1^ = 100 [ln (Wt/W_0_)]/t (2)

where Wt is the fresh weight biomass after t days in culture and W_0_ is the initial fresh weight biomass [17].

The productivity of the tanks was also calculated by the equation:

(g DW m^2^ day^−1^) = [(Nt − N_0_)/t(Dw/FW)]/A (3)

where (Nt − N_0_): weight difference in grams between weight at time (t) to the initial weight; t: time between Nt and N_0_; (Dw/FW): dry weight/fresh weight ratio [24].

As an indicator of the nutrient status of the biomass, total carbon (C) and nitrogen (N) content, and C:N ratios were determined by combusting the dried samples using a Flash EA 1112 Series Analyzer (Thermo Quest, Waltham, MA, USA) [58].

The average values of specific growth rate, productivity and C:N ratios were calculated for the experimental period and used for statistical analysis.

### 3.2. Sequential Extraction Process with a Biorefinery Approach

**Figure 4:**
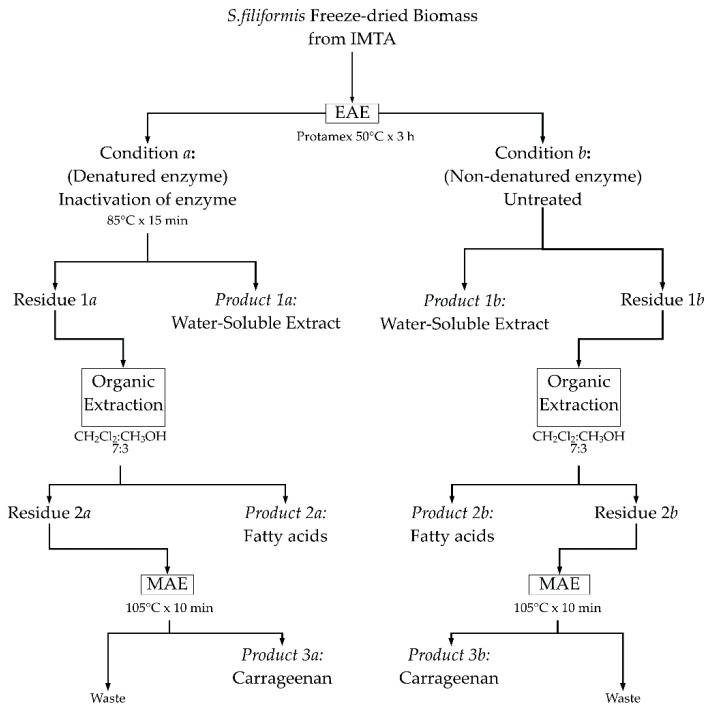
Schematic representation of the sequential extractions using a biorefinery approach.

Eco-friendly technologies were combined to propose achieve an integral utilization of *S. filiformis* biomass from IMTA using a three steps sequential extraction. Three valuable products (**Product 1**, **Product 2** and **Product 3**) were obtained (Figure 4).

The details of each extraction step is described as follows: the first step used an enzymatic assisted extraction (EAE) of *S. filiformis* biomass with protease enzyme (Protamex^®^). For the hydrolysis of seaweed, 200 mL of distilled water were added to 10 g of freeze-dried algal biomass, mixed with 5% *w*/*w* of protease, and placed in a 50 °C water bath for 3 h (a preliminary assay on the enzyme kinetics was performed to determine optimal time to maximize extraction yield, see Figure S1 in Supplementary Materials). The enzyme was then denatured (namely as condition ***a***) at 85 °C for 15 min. An enzymatic extraction without denaturing the enzyme (namely as condition ***b***) was also examined in order to evaluate the possible interference of gelling agents present in the algal cell wall that could be extracted due to heating when denaturing the enzyme. Blanks extracted under the same conditions but without enzymes served as controls (50 °C for 3 h + 85 °C for 15 min; 50 °C for 3 h, condition ***a*** and condition ***b***, respectively). After EAE, the extracts obtained in the different conditions were filtered and two fractions were obtained: a water-soluble extract (**Product 1*a*** and **Product 1*b***), and insoluble residues (Residue 1***a*** and Residue 1***b***). The **Products 1*a***–***b*** were freeze dried for further biochemical analyses (protein, carbohydrate, total lipid, and sulfate content), and for antiviral activity test. The insoluble residues were used as raw material for an organic extraction to extract lipids and fatty acids (**Product 2*a*** and **Product 2*b***). The dried residual mass after lipid extraction (Residue 2***a*** and Residue 2***b***) was finally used to obtain carrageenan (**Product 3*a*** and **Product 3*b***). For comparison, direct extractions (water soluble extract, fatty acid and carrageenan) of the initial biomass were also performed.

### 3.3. Carrageenan Extraction by Microwave-Assisted Extraction (MAE)

Carrageenan extraction was performed using a Microwave Accelerated Reaction System (MARS 800W, CEM, Matthews, NC, USA) at 100% of full power at a frequency of 2450 MHz. Briefly, 1 g of the dried residue (Residue 2***a*** and Residue 2***b***) was rehydrated overnight in 50 mL of distilled water. The sample was placed in a closed-vessel system (OMNI/XP-1500) to prevent solvent and analyte loss. By using closed vessels, the extraction can be performed at elevated temperatures accelerating the mass transfer of target compounds from the sample matrix. The internal temperature and pressure conditions were monitored within one reference OMNI/XP-1500 vessel equipped with temperature and pressure probes. The carrageenan extraction was performed at 159 kPa. After MAE, carrageenan was precipitated with 250 mL of Cetavlon (hexadecyl-tri-methylammonium bromide) in 9:1 (*v*/*v*) distilled water/acetone and recovered over filter paper in vacuum. The fibrous carrageenan was carefully washed three times with 63 mL of 95% ethanol saturated with sodium acetate to remove Cetavlon residues. Sodium acetate was removed by three final washes with 95% ethanol. Carrageenan was dried at 60 °C for 24 h, weighed to calculate percent yield, and milled to powder for further chemical (sulfate and 3,6 anhydro-galactose content) and structural analyses.

### 3.4. Chemical Analyses

Total soluble carbohydrates were measured by the phenol sulfuric acid method [59], and protein was determined with bovine serum albumin as a protein standard [60]. Sulfate content was determined by the barium chloride-gelatin turbidimetric assay [61], and 3,6 anhydro-galactose content was determined following the Matsuhiro and Zanlungo colorimetric method [62].

### 3.5. Lipid and Fatty Acids Extraction

Lipids were extracted with dichloromethane/methanol (7:3 *v*/*v*) for 24 h, simultaneously with maceration of the tissues assisted by mechanical agitation. The total lipid content was evaluated by the gravimetric method and reported as percentage of the algae dry weight [63]. The fatty acids from the total lipids were obtained by one-step direct transesterification method [64]. Briefly, 5 mg of lipids were treated with 5 mL of acetyl chloride/methanol (1:19 *v*/*v*) and esterified at 80 °C for 1 h. After cooling, 1 mL of water and 2 mL of n-hexane were added to the mixture, vortex, and centrifuged. The organic phases were collected, filtered, and dried with anhydrous sodium sulfate. Solvents were removed under nitrogen and the fatty acids methyl esters (FAMEs) solubilized in n-hexane were identified.

### 3.6. Gas Chromatography–Mass Spectrometry (GC–MS) Analysis

The FAMEs were analyzed by gas chromatography (Agilent, Model 7890B, Palo Alto, CA, USA) coupled with mass spectrometry (Agilent, Model 7000C, Palo Alto, CA, USA). FAMEs were separated on an HP-5MS capillary column (30 m × 0.25 mm, i.d., 0.25 μm film thickness), (Agilent J&W Scientific, Folsom, CA, USA). A sample (5 µL) was injected at temperature of 250 °C. Helium was used as the carrier gas at a flow rate of 1 mL min^−1^. The column temperature was programmed as follows: initial temperature at 100 °C for 4 min, ramps of 10 °C min^−1^ to 200 °C (for 5 min), and 10 °C min^−1^ to 300 °C for 15 min. Mass detector conditions were: electron impact mode at 70 eV, transfer line temperature 280 °C, source temperature 230 °C, mass acquisition range 50–500 amu (atomic mass units) and solvent delay 3.75 min. The FAMEs were identified by comparison of standard Supelco 37 Component FAME Mix (SUPELCO, St. Louis, MO, USA) and their mass spectra with those from the NIST/EPA/NIH Mass Spectral Library version NIST 2.2 (Agilent, Palo Alto, CA, USA).

### 3.7. Fourier Transform–Infrared (FT–IR) Spectra Analysis

The FT–IR spectra were recorded on a PerkinElmer Frontier FT–IR spectrometer (PerkinElmer FT-IR/Nir spectrophotometer Norwalk, USA), at room temperature. Carrageenan films were obtained by evaporation of an aqueous solution of carrageenan extracts (0.2% *w*/*v*) and measured in the infrared region between 4000 and 500 cm^−1^. The FT–IR spectra of commercial ι- and κ- carrageenans (Sigma-Aldrich, St. Louis, MO, USA) were also measured for comparison.

### 3.8. ^13^C Nuclear Magnetic Resonance (NMR) Spectra Analysis

For NMR analysis, 0.5 mg mL^−1^ of carrageenan in deuterium water was used. The spectra were recorded on a Varian/Agilent Premium Compact 600 NMR spectrometer at 70 °C, operating at frequencies of 150.81 MHz for ^13^C nucleus. The extracts were exchanged twice with 99.8% deuterium oxide (D2O) with intermediate lyophilization and dissolver in D2O (30 mg mL^−1^). Sodium [3-trimethylsilyl 2,2′,3,3′-2-*H*4] propionate (TSP-d4) was used as an internal reference for the baseline (0.00 ppm).

### 3.9. Determination of Antiviral Activity

The antiviral and cytotoxicity tests were determined in the water-soluble extracts (**Products 1*a***–***b***), and the carrageenans (**Products 3*a***–***b***) following the methodology described by Hardouin et al. [50].

#### 3.9.1. Cells and Viruses

African green monkey kidney cells (Vero ATCC CCL-81) were used for the antiviral test. The Eagle’s minimum essential medium (MEM, Eurobio, Courtaboeuf, France), supplemented with 8% fetal calf serum (FCS, Eurobio, Courtaboeuf, France) and 1% antibiotics PCS (10,000 IU mL^−1^ penicillin, 25,000 IU mL^−1^ colimycin, 10 mg mL^−1^ streptomycin; Sigma-Aldrich) was used as the culture medium. *Herpes simplex* virus type 1 (HSV-1) a wild-type strain 17 (sensitive to acyclovir) was provided by Prof. Agut (Laboratoire de Dynamique, épidémiologie et traitement des infections virales de la Pitié Salpêtrière, Paris, France). The culture was maintained at 37 °C under a 5% CO_2_ atmosphere, and the medium was renewed daily. The virus was multiplied by Vero cells infection, concentrated through centrifugation and stored at −80 °C until its use.

#### 3.9.2. In Vitro Antiviral and Cytotoxicity Evaluation by Cell Viability

The antiviral and cytotoxicity of the seaweed extracts were evaluated on the Vero cell/HSV^−1^ by incubating cellular suspensions (3.5 × 10^5^ cells mL^−1^) with five dilutions (concentration from 50 to 400 μg mL^−1^ for water soluble extracts (**Product 1**), and 1 to 200 μg mL^−1^ for carrageenan (**Product 3**) of fractions. The tests were performed in 96-well plates (72 h, 37 °C, 5% CO_2_) in Eagle’s MEM containing 8% FCS. For the antiviral assay, three replicates were infected using 50 μL of medium and a virus suspension at a multiplicity of infection (MOI) of 0.001 ID_50_ cells. After incubation, antiviral activity was evaluated by the neutral red dye method [21,64]. The antiherpetic compound acyclovir [9-(2-hydroxyethoxymethyl) guanine] was used as reference inhibitor. The 50% effective antiviral concentration (EC_50_) was expressed as the concentration that achieved 50% protection of virus-infected cells from virus-induced destruction, and the selectivity index (SI) values were calculated as CC_50_/IC_50._ The OD was related directly to the percentage of viable cells, which was inversely related to the cytopathic effect (CPE). The linear regression was determined for each assay on the basis of cell controls (0% CPE) and virus controls (100% CPE). Data were expressed as a percentage of protection (%P):

% P = [((ODt)virus − (ODc)virus)/((ODc) MOCK − (ODc)virus)] × 100(4)

where (ODt) virus was the OD (optical density) of the test sample, (ODc) virus was the OD of the virus-infected control (no samples), and (ODc) MOCK was the OD of the mock-infected control. The concentration of seaweed hydrolysate, which provided 50% protection to virus-infected cells, was determined from a dose-response curve using linear regression [65,66].

The cytotoxicity based upon cell viability was tested in triplicate using the neutral red dye method [64]. The positive control used was a Vero cellular suspension (3.5 × 10^5^ cells mL^−1^) in Eagle medium supplemented with 8% FCS and 1% antibiotics PCS. The cells were examined daily under a phase-contrast microscope to determine the minimum concentration of hydrolysate dry matter that induced alterations in cell morphology, including swelling, shrinkage, granularity and detachment. Absorbance was read using a multi-plate spectrophotometer (Packard Spectra Count™) at 540 nm. The 50% cytotoxic concentration (CC_50_) defined as the concentration that reduced the OD of treated cells to 50% of that of untreated cells, was calculated as:

CC_50_ = [(ODc) C − (ODc) MOCK/(ODc) C] × 100, (5)

where (ODc) C and (ODc) MOCK were the OD (optical density) values of the untreated cells and treated cells, respectively [66].

### 3.10. Statistical Analysis

Results were expressed as means ± standard deviation (SD). The concentrations of the extracts capable of killing 50% of the cells (CC_50_) were calculated by non-linear fit (GraphPad Prism 7 Software, La Jolla, CA, USA). The data was analyzed with student’s “t” tests or one-way analysis of variance (ANOVA) to determine if there were significant differences between treatments.

## Figures and Tables

**Table 1:** *Solieria filiformis* growth rate, productivity, C:N ratio and epiphyte content from an integrated multi-trophic aquaculture (IMTA) system and from the control unit (cultured only with seawater).

Culture System	Growth Rate (% day^−1^)	Productivity (g dw m^2^ day^−1^)	C:N	Epiphytes (%)
IMTA	12.0 ± 2.1 ^a^	26.2 ± 11.4 ^a^	23.8 ± 4.0 ^a^	3.3 ± 0.8 ^a^
Control	5.7 ± 1.7 ^b^	10.1 ± 4.8 ^b^	18.7 ± 2.1 ^a^	7.4 ± 0.9 ^b^

Different superscripts indicate significant differences (*p* < 0.05).

**Table 2:** Biochemical composition of *Solieria filiformis* from the IMTA system and the control unit (cultured only with seawater).

Culture System	Dry Weight (%)	Carbohydrates (%)	Protein (%)	Lipids (%)	Sulfate (%)
IMTA	12.68 ± 2.4 ^a^	22.47 ± 1.2 ^a^	8.83 ± 0.6 ^a^	2.49 ± 0.3 ^a^	18.29 ± 0.5 ^a^
Control	9.53 ± 1.3 ^b^	18.81 ± 1.6 ^a^	8.12 ± 0.9 ^a^	2.09 ± 0.1 ^b^	15.25 ± 0.5 ^b^

Different superscripts indicate significant differences (*p* < 0.05).

**Table 3:** Yield and biochemical composition of water-soluble extracts (**Products 1*a***–***b***) from *Solieria filiformis* and their residues (Residues 1***a***–***b***).

Enzyme-Assisted Extraction (EAE) (Extracts and Residues)	Yield (%)	Carbohydrates (%)	Sulfate (%)	Protein (%)	Lipids (%)
**Product 1*a***	72.4 ± 1.2 ^a^	11.6 ± 1.0 ^a^	6.1 ± 0.4 ^a^	12.3 ± 0.3 ^a^	0.72 ± 0.1 ^a^
**Product 1*b***	63.5 ± 2.1 ^b^	7.9 ± 0.7 ^b^	4.5 ± 0.2 ^b^	12.8 ± 1.3 ^a^	0.67 ± 0.1 ^a^
Residue 1***a***	19.9 ± 0.2 ^c^	32.3 ± 0.7 ^c^	24.4 ± 0.4 ^c^	4.6 ± 0.1 ^b^	0.71 ± 0.1 ^a^
Residue 1***b***	22.5 ± 0.1 ^d^	32.4 ± 1.7 ^c^	22.8 ± 2.1 ^c^	7.4 ± 0.5 ^c^	0.71 ± 0.1 ^a^

Different superscripts indicate significant differences (*p* < 0.05). ***a*** and ***b*** correspond to the EAE conditions with the denatured and non-denatured enzyme, respectively.

**Table 4:** Cytotoxicity and antiviral activity of water-soluble extracts (**Products 1*a***–***b***) from *Solieria filiformis*.

Product	CC_50_ (μg/mL)	EC_50_ (μg/mL)	Selectivity Index (CC_50_/EC_50_)
**Product 1*a***	>400	93.01 ^a^	4
**Product 1*b***	>400	>400 ^b^	1
Acyclovir	>400	3.2–5.4	74–125

Different superscripts indicate significant differences (*p* < 0.05). ***a*** and ***b*** correspond to the EAE with the denatured and non-denatured enzyme, respectively. Acyclovir: commercial product used to treat the infection by *H. simplex* virus type 1.

**Table 5:** Fatty acids (FAs) (% of total fatty acids methyl esters (FAMEs)) and nutritional indexes of **Products 2*a***–***b*** and in the initial *Solieria filiformis* raw biomass obtained by direct extraction.

FAs	Product 2*a*	Product 2*b*	FAs by Direct Extraction
*n*-C14:0	7.55 ± 0.56 ^a^	6.96 ± 0.64 ^a^	6.99 ± 0.01 ^a^
*n*-C15:0	3.73 ± 0.32 ^b^	3.06 ± 0.28 ^b^	1.64 ± 0.04 ^a^
*n*-C16:0	49.53 ± 6.63 ^a^	55.85 ± 8.23 ^a^	43.95 ± 4.79 ^a^
*n*-C18:0	8.38 ± 0.67 ^b^	7.69 ± 1.03 ^b^	3.13 ± 0.41 ^a^
**ƩSFAs**	**69.19 ± 8.18** ^a^	**73.56 ± 10.18** ^a^	**55.71 ± 5.24** ^a^
*n*-C16:1 ω7	3.73 ± 0.45 ^b^	6.12 ± 0.97 ^b^	21.28 ± 1.86 ^a^
*n*-C18:1 ω9 *	ND	1.4 ± 0.1 ^a^	3.54 ± 0.14 ^a^
*n*-C18:1 ω9 **	ND	1.56 ± 0.29 ^a^	3.29 ± 0.22 ^a^
*n*-C17:1	12.84 ± 1.21 ^b^	3.39 ± 0.32 ^c^	6.09 ± 0.94 ^a^
*n*-C18:1	ND	ND	1.76 ± 0.12
**ƩMUFAs**	**16.57 ± 4.94** ^b^	**12.47 ± 1.68** ^b^	**32.2 ± 3.28** ^a^
*n*-C18:3 ω6	ND	ND	1.57 ± 0.5
*n*-C18:2 ω6	ND	ND	1.41 ± 0.21
*n*-C20:4 ω6	ND	1.33 ± 0.23 ^b^	3.09 ± 0.55 ^a^
*n*-C20:5 ω3	ND	1.34 ± 0.14 ^a^	1.21 ± 0.3 ^a^
*n*-C16:2 ***	6.97 ± 0.89 ^b^	4.74 ± 0.89 ^c^	1.05 ± 0.3 ^a^
*n*-C16:2 ****	7.28 ± 0.89 ^a^	6.57 ± 0.97 ^a^	ND
**ƩPUFAs**	**14.25 ± 1.78** ^b^	**13.98 ± 2.23** ^b^	**8.33 ± 1.86** ^a^
**Ʃ** ω6	-	1.33	6.07
**Ʃ** ω3	-	1.34	1.21
**ω6/ω3**	-	**0.91**	**5.01**
**SFAs/UFAs**	**2.24**	**2.7**	**1.37**

ND = Not detected. * Oleic acid; ** Elaidic acid; *** 7,10-Hexadecadienoic acid; **** 9,12-Hexadecadienoic acid. ***a*** and ***b*** correspond to conditions with the denatured and non-denatured enzyme, respectively. Different superscripts indicate significant differences (*p* < 0.05).

**Table 6:** Yield and chemical characterization of *Solieria filiformis* carrageenan extracted from lipid extraction residues (**Products 3*a***–***b***).

Product	Carrageenan Yield (%)	Carbohydrates (%)	Sulfate Groups (%)	3,6 Anhydro-Galactose (%)
**Product 3*a***	17.09 ± 2.39 ^a^	34.34 ± 1.88 ^a^	18.15 ± 1.87 ^a^	18.57 ± 2.01 ^a^
**Product 3*b***	29.67 ± 4.14 ^b^	28.79 ± 1.02 ^b^	34.91 ± 3.65 ^b^	7.43 ± 0.77 ^b^
Carrageenan from direct extraction	25.55 ± 2.6 ^b^	22.47 ± 1.16 ^c^	47.66 ± 10.4 ^b^	16.11 ± 0.88 ^a^

Different superscripts indicate significant differences (*p* < 0.05). Percentage of carrageenan expressed as a function of the initial biomass. Carbohydrates, sulfate and 3,6 anhydro-galactose content are expressed as percentage of carrageenan yield. ***a*** and ***b*** correspond to conditions denatured and non-denatured enzyme, respectively.

**Table 7:** Cytotoxicity and antiviral activities of *Solieria filiformis* carrageenan (**Products 3*a***–***b***).

Product	CC_50_ (μg/mL)	EC_50_ (μg/mL)	Selectivity Index (CC_50_/EC_50_)
**Product 3*a***	>400	27.09 ^a^	15
**Product 3*b***	>400	16.15 ^b^	25
Carrageenan by direct extraction	>400	6.31 ^c^	63
Acyclovir	>400	3.2–5.4	74–125

Different superscripts indicate significant differences (*p* < 0.05). ***a*** and ***b*** correspond to conditions denatured and non-denatured enzyme, respectively. Acyclovir: Commercial product used to treat the infection by *H. simplex* virus type 1.

**Table 8:** Summary of yields and quality parameters (antiviral activity expressed as EC_50_ and polyunsaturated fatty acids (PUFAs) content) of *Solieria filiformis* products obtained by direct extraction and by integrated biorefinery process.

Products	Direct Extraction	Biorefinery (Condition *a*)	Biorefinery (Condition *b*)
**Water-Soluble Extract (WSE) yield (%) (Product 1)**	**63.5–72.4**	**72.4**	**63.5**
WSE EC_50_ (μg mL^−1^)	-	93.0	>400
Lipids (%)	2.49	0.7	0.7
**PUFAs (%) (Product 2)**	**8.33**	**14.25**	**13.98**
**Carrageenan yield (%) (Product 3)**	**25.51**	**17.09**	**29.67**
Carrageenan EC_50_ (μg mL^−1^)	6.31	27.09	16.15

## Supplementary Materials

The following are available online at http://www.mdpi.com/1660-3397/16/12/487/s1, Figure S1. Kinetics of Protamex extraction.
