# Supplementary figures and images for: Environmentally Friendly Valorization of Solieria filiformis (Gigartinales, Rhodophyta) from IMTA Using a Biorefinery Concept
Source: Mar Drugs. 2018 Dec 6;16(12):487. doi: 10.3390/md16120487 (PMC6315615; doi:10.3390/md16120487)

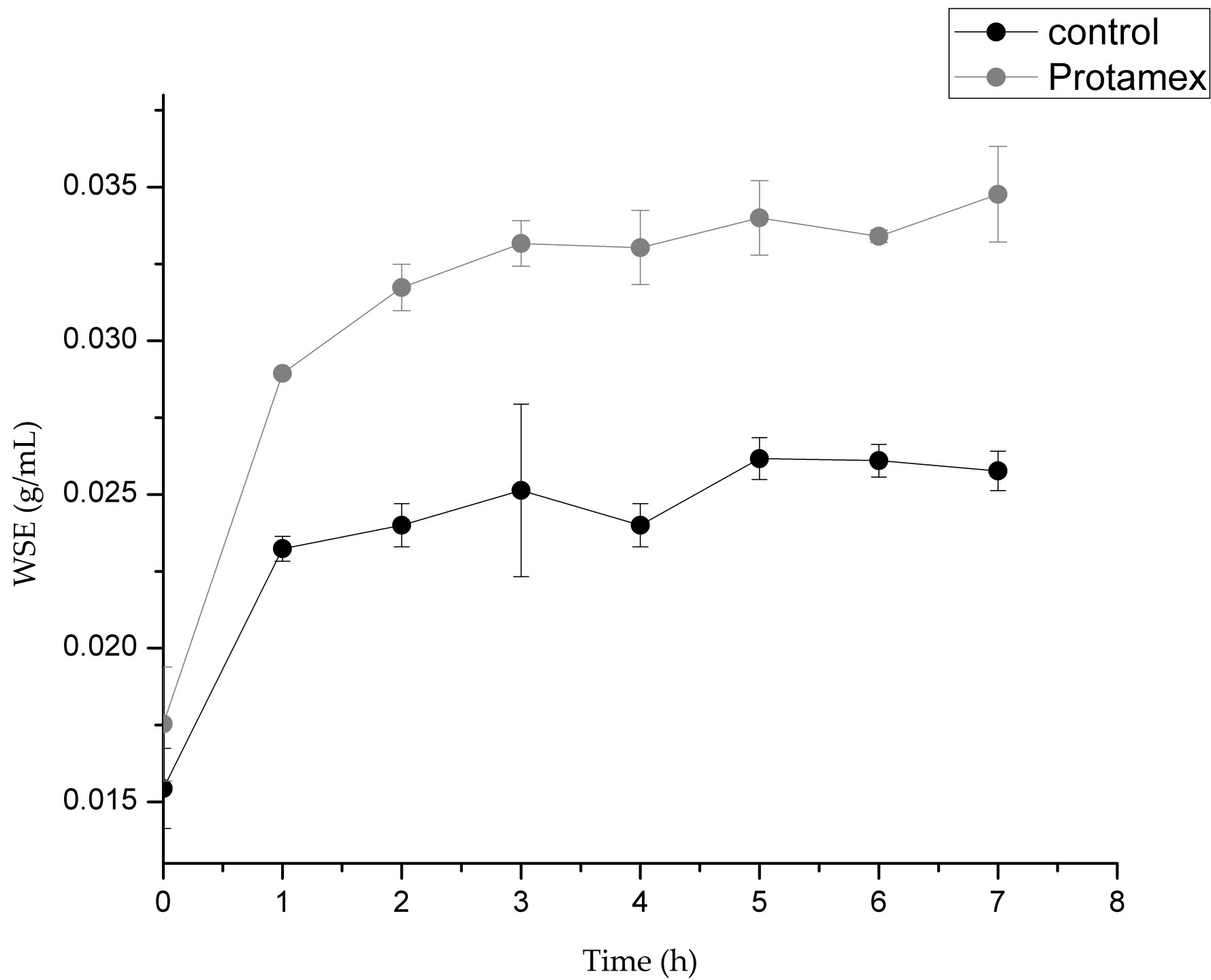

Supplement: Supplementary file 1 [file marinedrugs-16-00487-s001.pdf]
